# Zein as a Basis of Recyclable Injection Moulded Materials: Effect of Formulation and Processing Conditions

**DOI:** 10.3390/polym15183841

**Published:** 2023-09-21

**Authors:** Fahimeh Alsadat-Seyedbokaei, Manuel Felix, Carlos Bengoechea

**Affiliations:** Departamento de Ingeniería Química, Universidad de Sevilla, Escuela Politécnica Superior, 41011 Sevilla, Spain; fahsey@alum.us.es (F.A.-S.); cbengoechea@us.es (C.B.)

**Keywords:** bioplastic, injection moulding, rheology, tensile properties, zein

## Abstract

The growing concern about reducing carbon footprint has led to the progressive replacement of traditional polymeric materials by natural-based biodegradable materials. However, materials from natural sources (i.e., plants) typically possess poorer mechanical properties when compared to conventional plastics. To counterbalance this, they need to be adequately formulated and processed to eventually meet the standards for certain applications. Zein is the major storage protein from corn and can be obtained as a by-product from the corn-oil industry. It is an excellent candidate for producing green materials due to its stability, biodegradability, renewability, and suitable mechanical and technical-functional properties. In the present work, zein was blended with a plasticizer (i.e., glycerol) at three different zein/glycerol ratios (75/25, 70/30, and 65/25) and then injection moulded at three different processing temperatures (120, 150, and 190 °C). The properties of both blends and bioplastics were evaluated using dynamic mechanical analysis (DMA), tensile tests, and water absorption capacity (WUC). The properties–structure interrelation was assessed through a scanning electron microscope. Generally, a higher zein content and processing temperature led to a certain reinforcement of the samples. Moreover, all bioplastics displayed a thermoplastic behaviour finally melting at temperatures around 80 °C. The lack of massive crosslinking enabled this melting, which finally could be used to confirm the ability of zein based materials to be recycled, while maintaining their properties. The recyclability of thermoplastic zein materials widens the scope of their application, especially considering its biodegradability.

## 1. Introduction

The massive production of plastics and their ubiquitous presence in several commercial and industrial applications has become a significant threat to the environment [1]. Most of the plastic materials consumed nowadays are obtained from petrochemical sources, displaying attractive properties, such as lightweight, high mechanical strength, and easy processing [2]. Thus, they have been produced in large quantities since the 50 s and employed mainly for packaging applications, where most of the millions of tons produced every year (~85%) are disposed after use [3,4]. Common plastics, such as polyethylene, polypropylene, or polyvinyl chloride, are resistant to microbial degradation and remain in the environment for hundreds of years when discarded, negatively affecting the environment [5,6,7]. Moreover, fossil fuel resources are non-renewable, and their use by the plastic industry, even if smaller than other sectors (e.g., transport), contributes to the depletion of the existing resources. Additionally, the production and disposal of petrochemical plastics also result in emissions of carbon dioxide and toxic compounds, which also contribute to their global environmental impact [8]. As a consequence, research on biodegradable plastics produced by sustainable methods as an alternative has boosted in the last decades [9]. For some applications, bioplastics that are both bio-based and biodegradable are the best option to fulfil the requirements of future green plastic materials [3].

In this sense, proteins from different sources (i.e., soybean and wheat) are being investigated as potential raw materials to produce bioplastics for many applications such as food packaging or agriculture [10,11,12,13]. Corn is a very important crop, being the major cereal grain throughout the world [14]. Its industrial interest relies on the different fractions obtained, of which starch, the main component of the endosperm, is the one with the highest interest in the food industry. It is followed by the oil fraction extracted from the germ and by proteins, which are located in both endosperm and germ. Proteins can account up to 9–12% of the dry weight of corn seeds, existing different types of proteins: albumins and globulins (located mainly in the germ) and prolamin-type proteins (mostly found in the endosperm) [15]. Zein, the main prolamine protein found in corn, accounts for 45–50% of the total protein in corn [16] and, as it is deficient in essential amino acids for human nutrition (e.g., lysine, tryptophan), does not compete with the food supply chain [17,18]. Also, contrary to other proteins already investigated, such as albumen, soy, pea, or plasma [19,20], is a non-water soluble hydrophilic protein [21], which may be helpful for applications like coatings, plastics, textiles, or adhesives. Zein has already been investigated as raw material for the manufacture of bioplastics through casting [14,22] or extrusion [23,24]. However, studies on injection moulding are not so abundant [25] and not focused neither on the zein content nor the processing conditions, as in the present study.

The addition of plasticizers is required to process proteins adequately [26], as they reduce intermolecular interactions between protein chains, promoting the mobility of protein chains and, as a consequence, lowering the glass transition temperature (T_g_) [27]. Thus, the formulation of bioplastics should be modified depending on the mechanical strength pursued for every application. Glycerol (Gly) is the most used in the preparation of protein-based bioplastics [28].

The aim of this work has been to develop bioplastics from zein, using different zein/Gly ratios (65/25, 70/30, and 75/25) and three different moulding temperatures (120, 150, and 190 °C). Zein/Gly blends were initially homogenized in a mixer rheomether and then processed by injection moulding. Dynamic mechanical analysis (DMA) and tensile tests were performed to characterise the samples, as well as the determination of their water uptake capacity (WUC) and soluble matter loss (SML). The microstructure of probes was evaluated by scanning electron microscopy. Finally, the ability of zein materials to be recycled was assessed by characterising a reprocessed injection moulded sample.

## 2. Materials and Methods

### 2.1. Materials

Zein was provided by Shanghai Seasongreen Chemical CO (Shanghai, China) with a protein content of 92.4%. The protein content was determined in quadruplicate as % N × 6.25 using a LECO CHNS-932 nitrogen micro analyser (Leco Corporation, St. Joseph, MI, USA). Moisture, lipid, and ashes content was 6.1 ± 0.2, 2.0 ± 0.7, and 0.16 ± 0.01%, respectively. Pharma grade glycerol (Gly) was provided by Panreac Química S.A (Barcelona, Spain) and was used as plasticizer in all the formulations analysed in this research.

### 2.2. Sample Preparation

Bioplastic materials were obtained following a two-stage method. Firstly, zein and Gly were blended in a two-blade counter-rotating rheometer (Haake Polylab QC, ThermoHaake, Vreden, Germany) mixer at four protein/plasticizer levels (65/35, 70/30, 75/25, and 80/20). Higher protein contents were discarded as they led to a very solid dough-like material, whereas lower protein contents were too fluid to be processed by injection moulding. The mixer used allowed monitoring both temperature and torque during the overall mixing process, and dough-like blends were obtained after mixing for 10 min at 25 °C and 50 rpm.

Selected blends were then submitted to injection moulding to obtain the bioplastics probes using a lab-scale injection moulding machine MiniJet II (ThermoHaake, Vreden, Germany). Dumbbell and rectangular specimens (60 × 10 × 1 mm) were obtained using an injection pressure of 500 bar for 170 s and a holding pressure of 200 bar for 10s. The effect of the injection temperature was studied (from 120 to 190 °C), whereas mould temperature was kept always at 40 °C. For the recyclability of the materials obtained (Supplementary Figure S1), the probes were initially obtained as indicated and then cut into small pieces. The small pieces of material were reintroduced into the injection chamber and subjected to the above-mentioned processing conditions. New probes were obtained and characterized by tensile test experiments.

### 2.3. Methodology

#### 2.3.1. Dynamic Mechanical Thermal Analysis (DMTA)

Viscoelastic properties were determined for blends and bioplastics by small amplitude oscillatory compressional and tensional measurements, respectively. DMTA tests were performed in a DMA580 rheometer (TA Instruments, New Castle, DE, USA). Compression tests for blends were carried out in a 15 mm diameter cylindrical compression geometry, whereas rectangular tensile clamps were used for injection moulded bioplastics. Thus, frequency sweep tests were performed from 0.1 to 10 Hz on bioplastics. Moreover, temperature ramp tests were performed at 1 Hz and a heating rate of 5 °C/min from 20 °C to 150 °C for blends or from −30 °C to 110 °C for bioplastics. Strain sweep tests (0.001–10%) were carried out at 1 Hz before any measurement to determine the linear viscoelastic region. All frequency and temperature ramp tests were carried out within this linear viscoelastic region.

#### 2.3.2. Tensile Tests

Uniaxial tensile tests were performed at 25 °C at 0.01 mm·s^−1^ until reaching the breakdown of the probes using rectangular probes in a Universal Test Machine MTS (Eden Prairie, MN, USA). Stress-strain curves were obtained, and then typical mechanical parameters (Young’s modulus (E), maximum stress (σ_max_), and strain at break (ε_max_)) were obtained from these curves.

#### 2.3.3. Water Uptake Capacity (WUC)

The WUC of bioplastics was determined by drying the samples overnight in an oven at 50 °C (initial dry weight) and subsequently introducing samples into distilled water for 24 h (wet weight). Finally, the probes were submitted again to a drying process (final dry weight) by freeze-drying technology to maintain the microstructure of the samples. A LyoQest freeze-dryer (Telstar Technologies, Barcelona, Spain) was used at −80 °C and 1 Pa. WUC and loss of soluble material (LSM) were determined using the following equations:

(1) $$WUC\;\left(\%\right)=\frac{wet\;weight-final\;dry\;weight}{final\;dry\;weight}\times 100$$

(2) $$LSM\;\left(\%\right)=\frac{initial\;dry\;weight-final\;dry\;weight}{initial\;dry\;weight}\times 100$$

#### 2.3.4. Scanning Electron Microscopy (SEM)

The surface of the bioplastic samples after water immersion and subsequent freeze-drying was studied by SEM microscopy. A 10 nm Pd/Au layer was sputtered over the samples using a Leica AC600 coater (Wetzlar, Germany), and then they were observed with a Zeiss Evo microscope (Carl Zeiss Microscopy, White Plains, NY, USA). The samples were observed at 10 kV acceleration voltage and ×500 magnification.

### 2.4. Statistical Analysis

Measurements were carried out in triplicate. Uncertainty was expressed as standard deviations whose values were plotted in all the parameters calculated. Statistical analyses were carried out with t-tests and one-way analyses of variance (ANOVA) (*p* < 0.05) using STATGRAPHICS centurion 18 (version 18.1.12) software (Statgraphics Technologies, Inc., The Plains, Virginia, USA)

## 3. Results

### 3.1. Blends

#### 3.1.1. Mixing Stage of Blends

**Figure 1:**
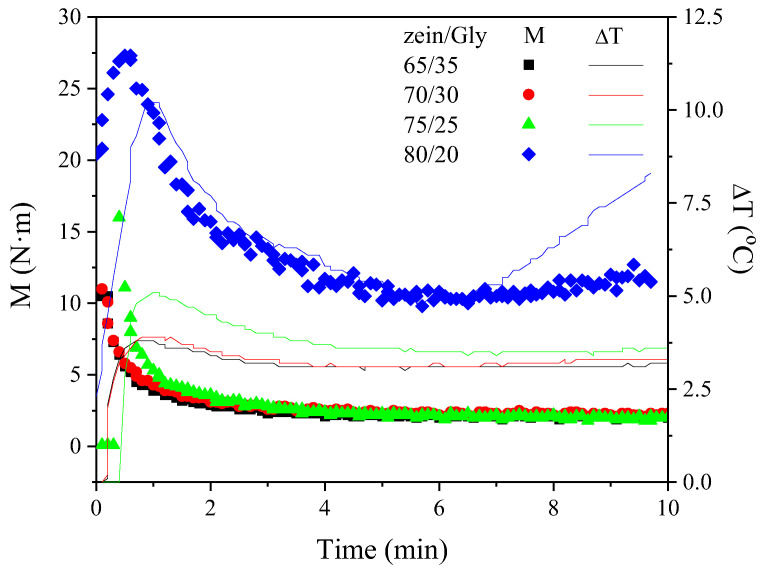
Torque and temperature increase evolution during the mixing of different zein/Gly blends: 65/35, 70/30, 75/25, and 80/20.

Figure 1 shows the evolution of torque and temperature increase over the 10 min mixing time of zein/Gly blends at four different ratios (65/35, 70/30, 75/25, and 80/20). A slight increase in torque value was observed at the beginning of the mixing stage, which was much more noticeable for the 80/20 ratio (reaching up to 28 N·m). This initial increase in torque is related to a former homogenisation of protein and plasticizer in the mixing chamber, and it is typically followed by a gradual decrease as zein and Gly blend together [29].

All the blends followed a similar pattern, regardless of the zein/Gly ratio, and reached an eventual torque around 2.5 N·m, except for the 80/20, whose final torque was more than four times higher than those displayed by the rest of the systems. This effect, together with the fact that the early maximum torque reached seemed to be directly related to the Gly content, may be related to the lubricant role played by the plasticizer that facilitates the biopolymer processability [13,30]. Thus, the lower the Gly content in the blend, the higher the torque reached either at the maximum or at the end of the mixing process.

The plasticizer effect exerted by Gly was also evidenced by the evolution of the temperature increase during mixing, since the higher the Gly content, the lower the temperature increased. This increase in temperature matched the torque dependence on the formulation and can be attributed to mechanical energy dissipation [31]. In fact, there are no significant differences within systems with the higher Gly content (i.e., 65/35 and 70/30), where the global temperature increase is minimal and around 3.2 °C. However, a noticeable increase in temperature was found for the 75/25 ratio (i.e., 5 °C) and was even more important for the 80/20 system (i.e., 10 °C). This result can be related to the energy dissipation as a consequence of the higher viscosity of the blends processed [20].

#### 3.1.2. Thermal Behaviour of Blends

**Figure 2:**
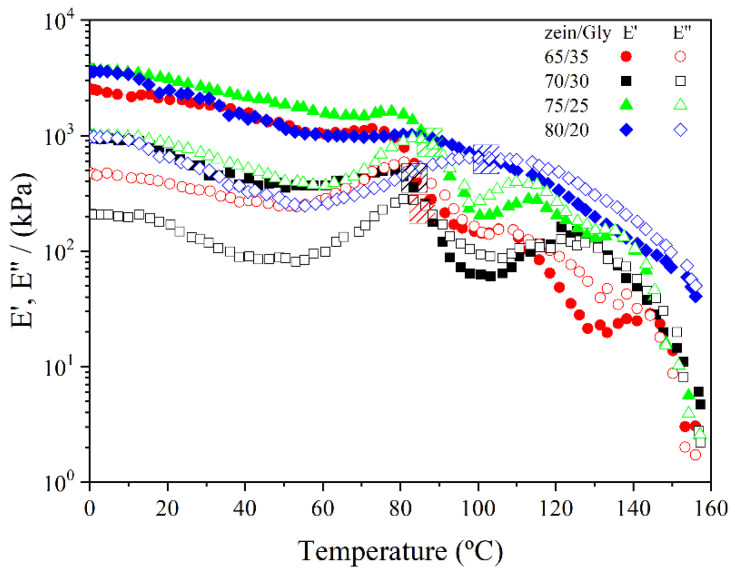
Evolution of viscoelastic moduli (E′ and E″) with temperature at 1 Hz of different zein/Gly blends: 65/35, 70/30, 75/25 and 80/20.

Figure 2 shows the temperature ramp tests performed on zein/Gly bends obtained at four zein/Gly ratios (65/35, 70/30, 75/25, and 80/20). The viscoelastic response of blends when heated is useful to assess their behaviour when submitted to the processing conditions in the injection moulding stage.

To adequately inject the blend into the mould cavities, the temperature should be initially high enough to enhance its flowability, so that the blend can easily flow through the die connecting the cylinder chamber to the mould cavity. However, once the blend is occupying the whole volume of the cavity, temperature should promote the fixation of the shape while degradation is inhibited [31,32,33].

At lower temperatures, a softening was detected as blends were heated through the decrease in the viscoelastic moduli, being initially the elastic modulus above the viscous. This thermoplastic behaviour during the initial phase of heating has been related to the enhanced mobility of the polymeric chains and typically found in other protein-based blends containing Gly as plasticizer, like soy or crayfish [20]. However, once temperature surpassed a certain value depending on the protein content (80–100 °C), blends acquired a predominantly viscous behaviour as the viscous modulus located then above the elastic moduli. This behaviour is different to that previously observed at high temperatures in other protein-based blends, where the elastic behaviour is reinforced as protein cross-linking takes [19,31,34,35]. Thus, blends containing zein exhibited an uncommon thermoplastic behaviour at high temperature, which can be linked to a reported glass transition temperature around 175 °C, as well as an endotherm thermal event related to protein relaxation [36]. DSC was performed to 75/25 blends and this temperature decreased to 128 °C, which agreed with the plasticizer effect of Gly and the result previously reported in literature.

### 3.2. Bioplastics

#### 3.2.1. Rheological Characterisation

The lack of reinforcement at higher temperatures in all blends inferred that these blends should be processed differently to other protein-based bioplastics which were thermoset in the mould. Thus, the thermoplastic behaviour shown by zein blends during the whole thermal treatment implies that samples should be cooled once injected in the mould, as it is frequent in thermoplastic synthetic polymers, such as polyethylene, polypropylene, and PVC among others [37,38]. This could be taken as an advantage for zein-based bioplastics as this processing method is well established industrially for synthetic polymers. Thus, the effect of both formulation (i.e., zein/Gly ratio) and processing conditions (i.e., cylinder temperature) was studied for zein-based bioplastics. Please notice that the 80/20 zein/Gly blend could not be injection moulded as its viscoelastic properties were higher than the rest, and its flowability was not enough to assure the complete filling of the mould cavity.

**Figure 3:**
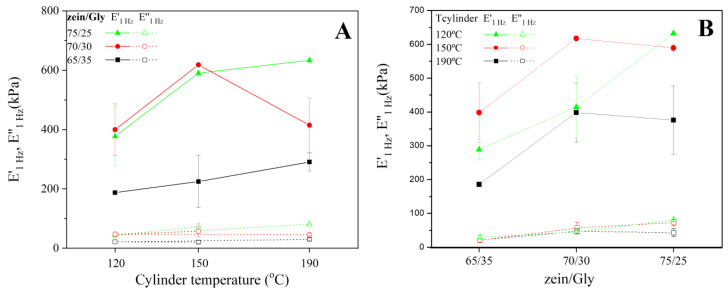
Viscoelastic moduli of bioplastics obtained at 1 Hz (E′_1_ and E″_1_, respectively) as a function of cylinder temperature (**A**) or zein/Gly ratio (**B**).

Figure 3A shows the viscoelastic moduli at 1 Hz (E′_1_ and E″_1_) obtained for zein/Gly injection moulded bioplastics at three cylinder temperatures (120, 150, and 190 °C). A clear effect of the processing temperature could be observed, as there was a significant increase in the viscoelastic moduli when the cylinder temperature increased from 120 to 150 °C in all cases (e.g., E′_1_ increased from around 380 to 600 MPa for the 75/25 zein/Gly bioplastic). The effect of temperature was also noticeable for the increase in the cylinder temperature from 150 to 190 °C, although in this case the increase in the viscoelastic moduli was not so relevant. The increase in the viscoaelastic properties when increasing the cylinder temperature could be due to the promotion of S-S exchange reactions [39] that reinforce the protein structure. Some authors reported a softening of a zein blend containing moisture once most cross-linking reactions have already occurred, which could explain the lack of reinforcement at the highest temperature [39]. Moreover, blends in this study included glycerol which exerts a different plasticizing effect than that of water which is a molecule much smaller than glycerol. Protein cross-linking promoted by temperature has previously been reported, observing a direct relationship between mould temperature and protein cross-linking [40].

When observing the effect of the zein/Gly ratio (65/35, 70/30, and 75/25) on E′_1_ and E″_1_ for bioplastics (Figure 3B), the higher the Gly content, the smaller E′_1_ values (e.g., E′_1_ decreased from 380 to 190 MPa when the zein/Gly ratio went from 75/25 to 65/35, respectively). This result agrees with previous results where the protein content of bioplastics ruled their mechanical properties [41]. However, increasing the zein content from 70/30 to 75/25 did not result in any significant differences (*p* < 0.05) when samples were processed at 120 or 150 °C. However, more drastic processing conditions (i.e., 190 °C for the cylinder temperature) led to a higher elastic modulus when zein content was higher.

**Figure 4:**
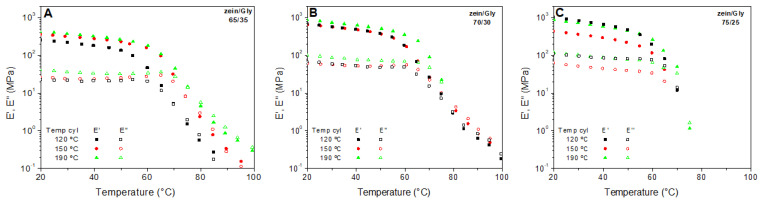
Dynamic mechanical thermal analysis (DMTA) of zein/Gly bioplastics at different cylinder temperatures (120, 150 and 190 °C) and zein/Gly ratios: 65/35 (**A**), 70/30 (**B**), and 75/25 (**C**).

Figure 4 shows the temperature ramp test performed on the zein-based bioplastics at three temperatures (120, 150, and 190 °C) and three zein/Gly ratios: 65/35 (Figure 4A), 70/30 (Figure 4B), and 75/25 (Figure 4C).

This figure shows a strong dependence of the viscoelastic moduli on temperature, where two regions could be detected for all samples: (i) below 60 °C, the elastic modulus was predominant and there was a weak dependence of the viscoelastic moduli on temperature; (ii) above 60 °C, the viscous modulus became predominant and an abrupt decrease of viscoelastic moduli with temperature could be distinguished and used to define a melting temperature (Tm) of the bioplastic (70.1 ± 4.5, 73.0 ± 2.4, and 69.1 ± 1.6 °C for 65/35, 70/30, and 75/25 ratios, respectively). A region similar to the first one at lower temperatures has been previously found in other protein-based bioplastics, such as crayfish and soy among others [27,42]. This region has been related to the mobility promoted by thermal agitation of polymeric molecules, which increased with temperature, leading to a decrease in the viscoelastic moduli [43]. However, the second region is not so typical for protein-based bioplastics, this response being similar to the melting behaviour of synthetic polymers such as polylactic acid (PLA) [44]. The melting of zein bioplastics at temperatures higher than 60 °C is an uncommon event for protein-based bioplastics, which could make the processing of these bio-based systems similar to common thermoplastic polymers, which may include traditional methods, such as injection moulding or extrusion, and other more innovative techniques, such as 3D printing. Note that this abrupt decrease in the viscoelastic moduli associated to the melting of the sample seemed to be delayed as the zein/Gly ratio increased. In any case, this effect is observed for all the systems regardless of the zein/Gly ratio (65/35, 70/30, or 75/25) or processing temperature (120, 150, or 190 °C)

#### 3.2.2. Tensile Tests

**Figure 5:**
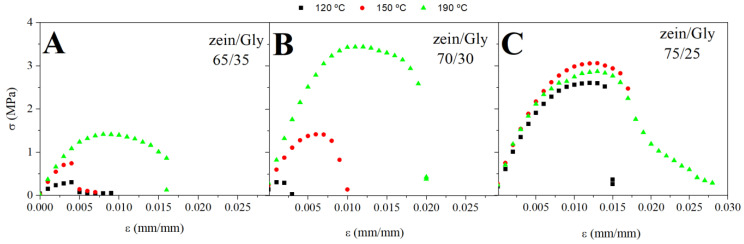
Stress-strain curves obtained for zein/Gly bioplastics obtained at different cylinder temperatures (120, 150 and 190 °C) at three zein/Gly ratios: 65/35 (**A**), 70/30 (**B**), and 75/25 (**C**).

Figure 5 shows the stress-strain curves obtained from tensile tests for bioplastics obtained at three zein/Gly ratios (65/35, 70/30 and 75/25) and three cylinder temperatures (120, 150 and 190 °C). The behaviour observed for all bioplastics was characteristic of polymeric materials [23] and was defined by an initial linear rise in stress as the materials were deformed at constant rate (i.e., elastic deformation). Then, a region characterised by stress decay as deformation continued (plastic deformation) appeared. Finally, a neck can be formed (in some cases) in the middle of the probes, leading to an abrupt drop in the tensile stress, and then a ductile rupture was detected some cases [45].

**Figure 6:**
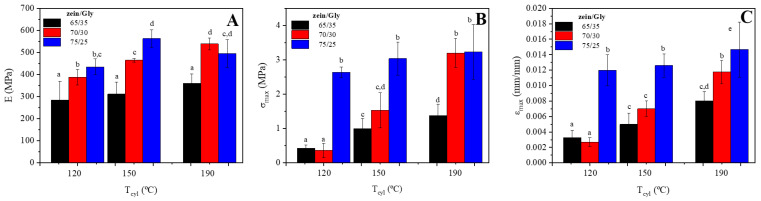
Mechanical parameters (E (**A**), σ_max_ (**B**), and ε_max_ (**C**)) obtained from stress-strain curves for ZPI/GL bioplastics obtained at different cylinder temperatures (120, 150, and 190 °C) at three ZPI/GL ratios (65/35, 70/30, and 75/25). Different letters above bars indicate significant differences (*p* < 0.05).

Figure 6 shows the effects of both formulation and processing temperature on the typical mechanical properties: Young’s modulus €, tensile strength (σ_max_), and strain at failure (ε_max_).

The effects of bioplastic formulation on the viscoelastic properties of the bioplastics were confirmed by the display of mechanical properties since an increase of Gly content in the formulation involved a decrease in the σ_max_ of the bioplastic. Moreover, the processing temperature also had an effect on the mechanical properties of the bioplastics since an increase in cylinder temperature resulted in an increase in both σ_max_ and ε_max_. A correlation between E and the zein/Gly ratio could be evidenced in Figure 6A, as an increase of zein content in the formulation involved an increase of E, implying that a greater protein content lead to a reinforcement of the materials, which became more rigid. Moreover, higher cylinder temperatures generally resulted in an upwards evolution of E values, although differences were much smaller between 150 and 190 °C, which could be related to a balance between S-S exchange reactions and softening of the reinforced structure [39].

Figure 6A,B show similar effects of formulation and processing temperature on both σ_max_ and ε_max_, respectively. Thus, an increase in the zein content or cylinder temperature involved an increase in the strength and deformability of the samples. Again, the effect of temperature was less relevant for the higher zein/Gly ratio (75/25). The σ_max_ of the materials obtained are lower than those typically found for some synthetic polymers such as LDPE and PA [46]; however, this parameter is similar to the one obtained for the PVC foam HD130 [47]. Thus, these results confirmed the effect of protein content on the mechanical properties of bioplastics, as a higher content of protein resulted in strengthened materials able to resist larger deformations. This effect has been already observed in other bioplastics based on proteins, where glycerol content can be modified to obtain the desired mechanical properties [48].

#### 3.2.3. Water Uptake Capacity

**Figure 7:**
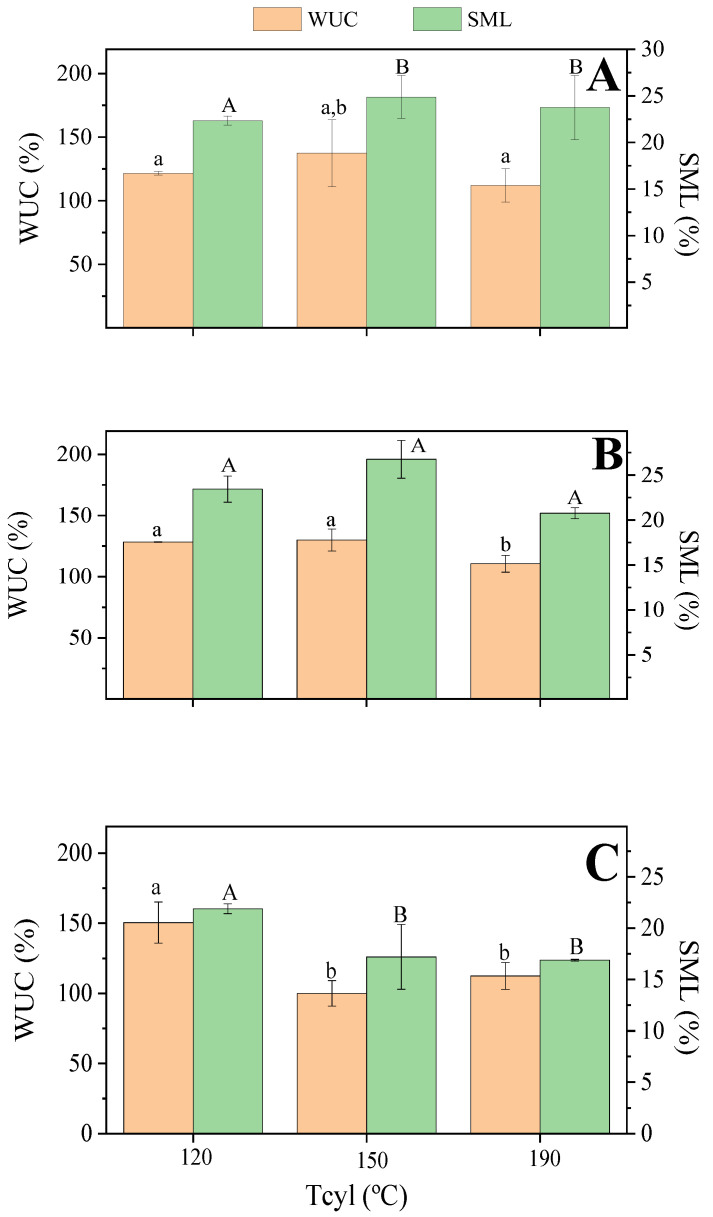
Water uptake capacity (WUC) and soluble matter loss (SML) for ZPI/GL bioplastics obtained at different cylinder temperatures (120, 150, and 190 °C) at three ZPI/GL ratios: 65/35 (**A**), 70/30 (**B**), and 75/25 (**C**). Different letters above bars indicate significant differences (*p* < 0.05).

Figure 7 shows the water uptake capacity (WUC) and soluble matter loss (SML) values after 24 h water immersion for bioplastics processed at different cylinder temperatures (120, 150, and 190 °C) and at zein/Gly ratios (65/35 (Figure 6A), 70/30 (Figure 7B), and 75/25 (Figure 7C)).

The effect of cylinder temperature on the WUC of the bioplastics depended on the zein/Gly ratio. Thus, the increase from 120 to 150 °C did not result in any significant effect on WUC for bioplastics with a zein/Gly ratio equal to 65/35 or 70/30 and remained around 130% in both cases. This agrees with the limited effect of temperature observed on the mechanical properties of those systems. However, WUC decreased from around 150 to 110% when the zein/Gly ratio was 75/25, which could be attributed to restricted swellability due to the promotion of biopolymer cross-linking when increasing the processing temperature [49]. This effect is even more noticeable at the highest cylinder temperature (i.e., 190 °C), where the decrease in WUC was noticeable in all cases. As for the effect of the plasticizer content on WUC values, the only differences are observed at the lowest processing temperature analysed (i.e., 120 °C), where the increase of zein involved an increase in the WUC.

Regarding the SML, zein is considered a non-water-soluble protein fraction, and consequently, the only soluble matter is expected to be Gly. This should be related to the fact that temperature exhibited a small effect on SML, as even if protein cross-linking was promoted by temperature, protein solubility in water was rather low in every case. By contrast, the decrease in glycerol content (i.e., higher zein/Gly ratio) resulted in a decrease in SML, as the content of soluble mass was lower. The observed effect of plasticizer content on SML has already been reported for protein-based bioplastics since processed protein-based bioplastics display generally a low-solubility and the plasticizer is mostly the solubilized component in these tests [50]. In any case, the above-mentioned protein cross-linking was also reflected in the SML estimations since an increase in cylinder temperature involved a decrease in the SML, which was more evident at 75/25 zein/Gly ratio.

#### 3.2.4. Morphology (Scanning Electron Microscopy)

**Figure 8:**
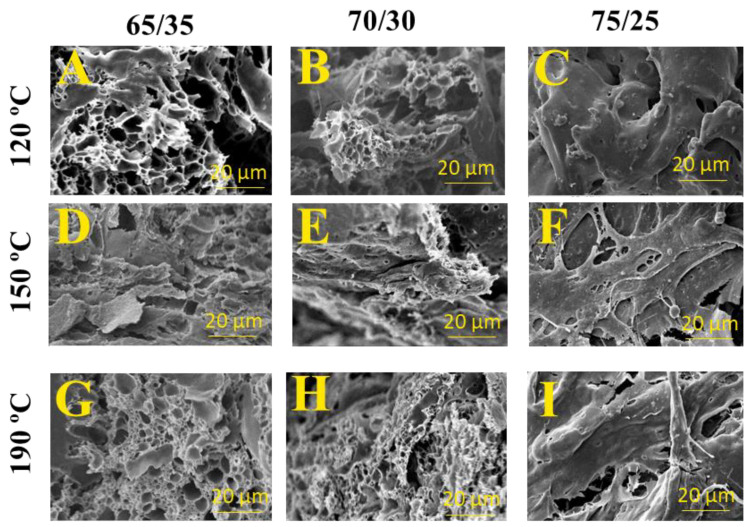
SEM images obtained for zein/Gly bioplastics at three cylinder temperatures: 120 °C (**A**,**B**,**D**), 150 °C (**D**,**E**,**F**), and 190 °C (**G**,**H**,**I**); and three zein/Gly ratios; 65/35 (**A**,**D**,**G**), 70/30 (**B**,**E**,**H**) and 75/25 (**C**,**F**,**I**).

Figure 8 shows SEM micrographs after 24 h water immersion of the different bioplastics obtained. In general, porous structures were obtained in all cases, which would indicate that glycerol was easily released during this water immersion stage, and consequently water was able to penetrate through the microstructure of the bioplastics. The formation of interconnected paths from the surface to the interior of the materials is essential for water uptake since it allows probes to absorb water and swelling [51]. Porous microstructure after water absorption is typical for other bioplastic materials, which could enable the re-absorption of water in a second immersion stage [52,53,54].

#### 3.2.5. Recyclability Potential of Zein Bioplastics

Considering the observed melting for bioplastics, the ability of zein materials to be melted and then re-processed through injection moulding was assessed. For that purpose, the bioplastic formulated with a zein/Gly ratio of 75/25 and injection moulded at 190 °C was selected, cut into pieces, and then fed to the cylinder of the injection moulding device, employing the same conditions as before. Zein-based bioplastics were re-processed for five times and subsequently characterised, leading to virtually comparable properties as the original sample (Supplementary material). This ability of zein materials to be potentially recycled is linked to the displayed thermoplastic behaviour, as thermoplastic polymers can be melted and reformed and then easily recycled. Even if their properties typically degrade with each reuse, results obtained prove that, at least, zein materials remain unaltered after one recycling step. Most protein systems behave as thermosetting polymers when heated at temperatures as high as those employed in this study, being much more difficult to recycle. In any case, to the best of our knowledge, the ability of protein-based materials to be recycled while keeping their properties has not yet been reported suggesting initial tests this possibility for zein-based bioplastics.

## 4. Conclusions

Bioplastics based on zein were successfully developed by injection moulding using glycerol as plasticizer. The presence of the plasticizer was required in the formulation as it enabled the processability of the materials since no bioplastics could be satisfactorily obtained for the highest protein content studied (80/20 zein/Gly). Even if most proteins typically go through crosslinking when heated, zein exhibited an uncommon behaviour since it behaved as a thermoplastic polymer, which melted above c.a. 80 °C, which resembles the behaviour of TPS-based materials. Thus, zein bioplastics required a relatively low mould temperature to fixate the shape of the final bioplastic, contrary to most protein systems. However, the properties of the bioplastics processed by injection moulding at 65/35, 70/30, and 75/25 zein/Gly ratios were comparable to those of for other protein-based bioplastics. In this sense, both the cylinder temperature and, especially, the zein/Gly ratio influenced the viscoelastic, mechanical, and water uptake of the bioplastics obtained. Thus, the maximum stress increased 1.5 times when the zein content increased from 65 to 75% in the formulation of bioplastics processed at 120 °C. However, if processed at 190 °C the increase was lower (i.e., 1.3 times). Temperatures above a certain melting temperature led to a fluid material as the biopolymer mobility increased. If crosslinking was promoted at higher temperatures, it was not certainly to a large extent as no phase change from liquid to solid state could be observed. Moreover, an increase in the amount of zein led to an increase in water absorption, being most of the materials loss during water immersion related to the plasticizer content, as zein is mostly non soluble in water. The loss of glycerol resulted in the formation of a self-supporting porous structure, which could be interesting for applications where aqueous solutions are to be absorbed while the structure of the material is to remain unaltered during their service life.

More importantly, the ability of zein materials to be recycled was assessed by five re-processing cycles. These initial tests confirm the possibility of re-processing the initial bioplastic probes since virtually comparable properties as the original sample were obtained. Even if future work needs to be carried out to determine important parameters, such as number of times samples can be recycled, these findings are of extreme importance when assessing the potential of zein bioplastics on an industrial scale.

## Data Availability

Data available on request from the authors.

## Supplementary Materials

The following supporting information can be downloaded at: https://www.mdpi.com/article/10.3390/polym15183841/s1, Figure S1: Mechanical properties of the re-processed zein-based bioplastics.
