# Supplementary material for: Zein as a Basis of Recyclable Injection Moulded Materials: Effect of Formulation and Processing Conditions
Source: Polymers (Basel). 2023 Sep 21;15(18):3841. doi: 10.3390/polym15183841 (PMC10535502; doi:10.3390/polym15183841)
Supplement: Supplementary file 1 [file polymers-15-03841-s001.zip › polymers-2624424-supplementary.pdf]

Supplementary Materials

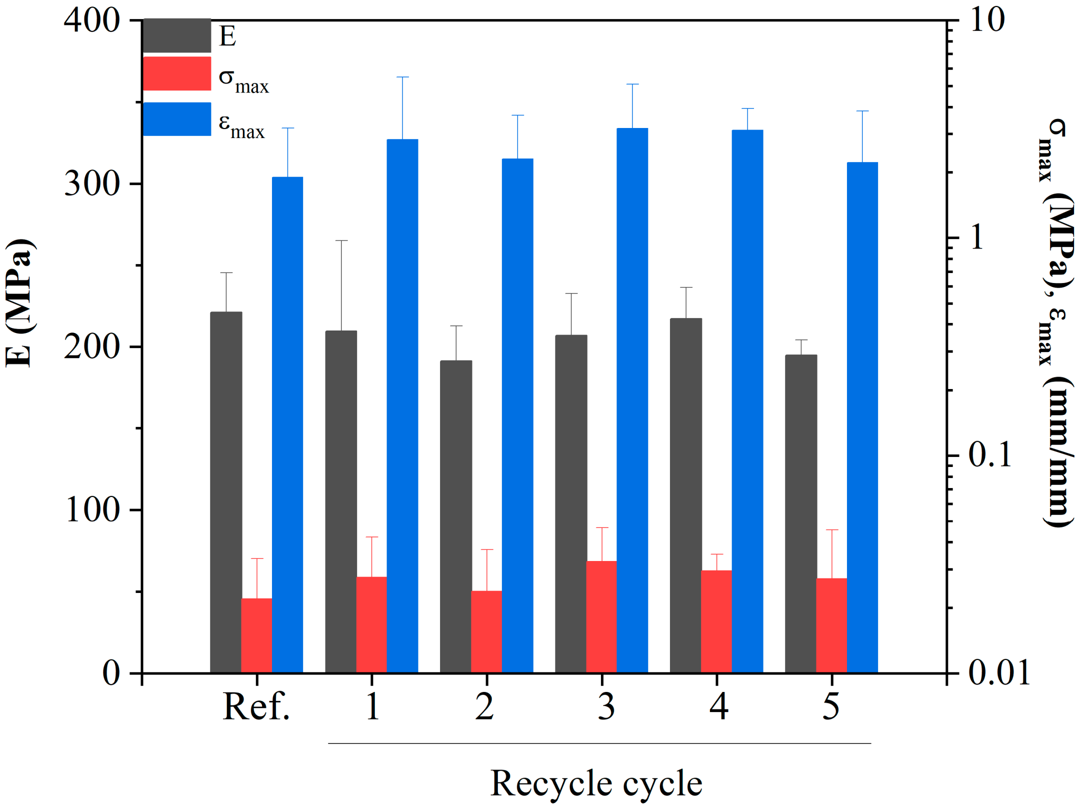

Figure S1. Mechanical properties of the re-processed zein-based bioplastics.
